# Assessment methodology for human-exoskeleton interactions: Kinetic analysis based on muscle activation

**DOI:** 10.3389/fnbot.2022.982950

**Published:** 2022-10-26

**Authors:** Vasco Fanti, Vittorio Sanguineti, Darwin G. Caldwell, Jesús Ortiz, Christian Di Natali

**Affiliations:** ^1^Department of Advanced Robotics (ADVR), Istituto Italiano di Tecnologia (IIT), Genova, Italy; ^2^Department of Informatics, Bioengineering, Robotics and Systems Engineering (DIBRIS), Università degli Studi di Genova (UniGe), Genova, Italy

**Keywords:** assistance, emg-to-force processing, exoskeletons, human-robot interaction, kinematic, kinetic

## Abstract

During the development and assessment of an exoskeleton, many different analyzes need to be performed. The most frequently used evaluate the changes in muscle activations, metabolic consumption, kinematics, and kinetics. Since human-exoskeleton interactions are based on the exchange of forces and torques, the latter of these, kinetic analyzes, are essential and provide indispensable evaluation indices. Kinetic analyzes, however, require access to, and use of, complex experimental apparatus, involving many instruments and implicating lengthy data analysis processes. The proposed methodology in this paper, which is based on data collected *via* EMG and motion capture systems, considerably reduces this burden by calculating kinetic parameters, such as torque and power, without needing ground reaction force measurements. This considerably reduces the number of instruments used, allows the calculation of kinetic parameters even when the use of force sensors is problematic, does not need any dedicated software, and will be shown to have high statistical validity. The method, in fact, combines data found in the literature with those collected in the laboratory, allowing the analysis to be carried out over a much greater number of cycles than would normally be collected with force plates, thus enabling easy access to statistical analysis. This new approach evaluates the kinetic effects of the exoskeleton with respect to changes induced in the user's kinematics and muscular activation patterns and provides indices that quantify the assistance in terms of torque (AMI) and power (API). Following the User-Center Design approach, which requires driving the development process as feedback from the assessment process, this aspect is critical. Therefore, by enabling easy access to the assessment process, the development of exoskeletons could be positively affected.

## 1. Introduction

The past 30 years have seen an exponential growth in interest in exoskeletons and wearable robotics, Bao et al. (2019). Application domains include but are not limited to medical and rehabilitation applications (Chen et al., 2013; Huo et al., 2016), military equipment (Jia-Yong et al., 2020), and industrial operations (Crea et al., 2021). In rehabilitation, exoskeletons can be used to treat different pathologies (Chang and Kim, 2013; Frisoli, 2018; Molteni et al., 2018) and therefore they may be active, passive, resistive, or interactive devices depending on the situation. In military applications (Murugan, 2021), the exoskeletons are often intended to reinforce the power/endurance of soldiers to allow them to carry heavy loads for extended periods, reducing their fatigue. In occupational applications, the primary motivation behind industrial exoskeletons is to prevent musculoskeletal disorders and reduce the risk of injury (Peters and Wischniewski, 2019).

Referring to exoskeletons, the physical human-robot interaction takes the significance of force transfer to empower and overcome human physical and motoric limits (Alami et al., 2006; Pons, 2010). The key characteristic of exoskeletons is the close physical interaction between the user and the robotic device during typical tasks, which often are repetitive and cyclical. Therefore, the device assessment and the evaluation of the efficiency and efficacy of the exchange of forces are essential. A positive interaction between the human and the robot could lead to important physical improvements (Nolan et al., 2018), while a poor exchange could lead to harmful consequences (e.g., skin irritations, soft tissue injury, falls, etc.) (Rathore et al., 2016; He et al., 2017). Exoskeletons used for gait assistance (Lovrenovic and Doumit, 2016; Young and Ferris, 2017; Cao et al., 2021) are a typical example of cyclical interaction due to the need to support the walk at every step.

To evaluate the human-robot interactions, exoskeleton performances and effects are commonly compared against a baseline condition, which is usually when the exoskeleton is not worn (Barbareschi et al., 2015). Typically assessments aim to evaluate changes in motion patterns, in muscle activation patterns, and in the characteristic physical forces associated with the specific task. In particular, the assistance is typically assessed by considering one or more of the following: muscle activation, kinetics, motion, and metabolic consumption analysis (Pinto-Fernandez et al., 2020; Pesenti et al., 2021). Focusing on kinetic analysis, the aim is to investigate the internal and external exchange of forces, and as a consequence, to investigate the articular torques and powers. The gold standard method for kinetic analysis relies on force plates and motion systems, and, by applying inverse dynamics, investigates the joint torque and power (Silva and Ambrósio, 2002). This analysis is essential, however, it is not always feasible. If the task does not involve any dynamic activity such as walking, the study can be easily performed *in situ*. Otherwise, when the task is dynamic, the data collection could be extremely complex or impossible. To accurately record data, force plates need to be pressed by the entire footprint, but this can alter the subject's natural walk (Shahabpoor and Pavic, 2017), create measurement errors, and lead to invalid results. The subjects could be tempted to increase or decrease their stride length thus, to affect the dynamic pattern and reducing the set of reliable data. Moreover, when using force plates only a single step can be recorded for each trial unless a substantial number of force plates or a treadmill with force sensors embedded are available.

Different approaches to kinetic analysis have been pursued by Lloyd and Besier (2003), Lenaerts et al. (2008), and Heintz and Gutierrez-Farewik (2007), using techniques based on EMG to force processing or static optimization. Despite their validity and accuracy, all these models involve the use of force sensors (Hashemi et al., 2015; Pizzolato et al., 2015). The method proposed in this paper, on the opposite, does not use force sensors even for the calibration phase and yet manages to have a low computational impact. Furthermore, compared to other methods that assess the performance of exoskeletons through the use of dummies (Nabeshima et al., 2018) for standardization of the evaluation process, our proposed method places the human operator at the core of the human-robot interaction. In fact, we propose a holistic approach using indices that put together kinetic and muscular factors, providing a comprehensive evaluation of the exoskeleton performances and its' impact on the final user.

The key features of this work are improvements in, and simplification of, the accessibility to such analysis and related outcomes, thereby providing a fast evaluation technique that can be used both to guide the design development process and fine-tune the control of the exoskeleton. This approach, based on a simplified musculoskeletal model of the joint under analysis, calculates the dynamic parameters using only measurements of the muscular activations (Hof and Van den Berg, 1981; Bogey et al., 2005) and joint rotations (Bogey et al., 2010; Dorschky et al., 2019). Our proposed method, when combined with an iterative process of exoskeleton development (such as the user-centered design approach suggests), could optimize the outcome resulting from the iterative approach phases, speeding up the device performance validation (ISO-9241, 2010) while relying on an approach based on statistical considerations due to the large number of cycles recorded. It generates a rapid calculation of the joint moments/torques and powers with specific comprehensive indices called *Assistive Moment Index* (AMI) and *Assistive Power Index* (API), providing direct information on the interaction with the exoskeleton and the consequent effects on the muscular and the joint motion patterns.

To assess and show the potential of this new method, the performance of a quasi-passive exoskeleton for walking assistance is evaluated, and a dedicated control strategy has been developed. A full performance assessment was conducted using seven healthy subjects. The hypotheses of the methodology validation are: i) the reproduction of joint kinetics signals from muscle activities optimization by obtaining at least 90% correlation coefficients with the signals used for the calibration; ii) verifying that the signals gathered from the literature have at least 85% correlation with the signals derived in the laboratory by inverse dynamics. The hypotheses for the assessment phase are: iii) the creation of evidence through specific indices for assessing exoskeletons; vi) verifying effective reduction of torque and/or power at a specific joint, in conjunction with muscular activity reduction during the walking task; v) evaluating the increment of torque and/or power or muscular activity at a specific joint, as a side effect of the user's interaction with the exoskeleton.

## 2. Methods

### 2.1. Human-exoskeleton interaction and assistance evaluation

When developing exoskeletons and wearable robotics the interaction between the user and the device is of the highest priority. These physical interactions can range from very simple, such as adding a mass to a walking person (Bastien et al., 2005; Jin et al., 2017) or loading elastic straps while wearing passive devices (Van Dijk and Van Der Kooij, 2014), to more complex actions that occur while wearing actively and quasi-passively actuated devices in dynamic tasks (Banala et al., 2007; Van Dijk et al., 2017; Di Natali et al., 2019). In all cases, there is a change in the kinematic and/or muscular patterns (Hidler and Wall, 2005). Considering active devices, the forces are generated by motors and transferred to the user as torques. It is therefore expected that less muscle activation occurs when the joint torques of the user and the exoskeleton are in agreement, and greater muscle activation when they work against each other. In passive and quasi-passive devices, users have to transfer the energy into the actuator before they could receive it back as a torque. Operationally the two phases are called *energy storing* and *releasing* (Di Natali et al., 2020). With elastic and spring-based devices, greater muscular activation occurs during the storage and there is less activation during the release of the passive elements. In all active, passive, and quasi-passive exoskeletons, the muscular activation profile when the exoskeleton is worn (*Exo* condition) can be very different from the corresponding baseline condition without the exoskeleton (namely *Noe*). Making movements under the influence of external forces may cause changes in muscle activation, and consequently, may lead to different motion and muscular patterns. Suppose the same movement is performed with and without the exoskeleton. In that case, it is postulated that if the muscular activation, and consequently the muscle force, in the *Noe* condition is greater than the *Exo*, then the interaction between the user and the exoskeleton is providing assistance to the muscle. Conversely, if the activation recorded in the muscle in the *Exo* condition is greater than in the *Noe*, the muscle is making more effort. In the first case, with muscle activation in the *Noe*>*Exo*, it can be hypothesized that the reduction in activity is due to the external energy that promotes movement and thus brings assistance to the user. When the activation in the *Noe*<*Exo*, the situation is not quite so straightforward. In fact, this cannot definitely be traced to resistance to joint movement alone, but could involve several other factors (stiffening due to body weight support, balancing forces, internal dissipation elements, compensative effects, intermuscular synergies, etc.) (Zelik and Kuo, 2010) and therefore analyzes of these results are more complex. For these reasons, it is possible to highlight the task phases during which the muscle receives assistance and quantify it, but the opposite is not possible.

Moreover, the assistance can no longer be investigated by only analyzing muscular aspects but requires analyzes of joint kinetics. Exoskeletons, in fact, work by sharing torques with the users, and, consequently, this could lead to changes in their joint moments and power. Typical evaluations compare joint torque and power in the *Exo* configuration, with the same data recorded in the *Noe* condition. In this case, the assistance depends on the task objective. If the purpose is to improve kinetic performance, it is desirable to obtain higher torque and power values in the *Exo* condition; if the aim is to reduce or re-modulate the user's kinetic contribution, lower activation levels for both the torque and the power are desirable while wearing the device.

In all cases, it is critical to perform an analysis with high statistical validity by evaluating the changes induced by the exoskeleton during multiple experimental cycles.

### 2.2. Index-based assistive methodology

The proposed methodology allows the calculation of kinetic parameters starting from muscular activations. As with torque, power, and kinematics in general, muscle activity is a crucial data point to be monitored during any assessment of an exoskeleton because it is directly influenced by the physical interaction between the device and the subject under analysis. Monitoring muscle signals during the design phase of the control strategy allows for a direct understanding of the interactions with the robot, and consequently enhances fine-tuning of the control strategy. Moreover, and very importantly, this method benefits from the ability to relatively easily collect a large sample of data (seven subjects for 10 min walking collected more than 10,000 steps with the exoskeleton and the same amount without it) while simultaneously, reducing the complexities and costs of the experimental apparatus, and time needed to perform the related data analysis. Finally, this tool allows simultaneous kinetics and muscular assistance analysis by providing specific indices, namely AMI and API, that quantify the torque and power exchange occurring during the human-robot interaction.

### 2.3. Overview

As introduced in Section 2.1, by analyzing muscle activity it is possible to identify the phases of a task during which the muscle is benefiting from the interaction with the exoskeleton and to quantify the benefit in terms of muscle activation and thus force delivered. For joint performances, on the other hand, it is possible to obtain information about the articular kinetics in terms of overall torque and power. Combining these features it is possible to have a mixed assistance index that considers both the joint and the muscle contributions. To perform the analysis in a proper way it has to be noted that: (i) the EMG and kinematic data have to be recorded in both the *Exo* and the *Noe* configurations; (ii) to analyze the overall interactions with the exoskeleton the activity of both agonistic and antagonistic muscles acting on the target joint must be recorded.

**Figure 1:**
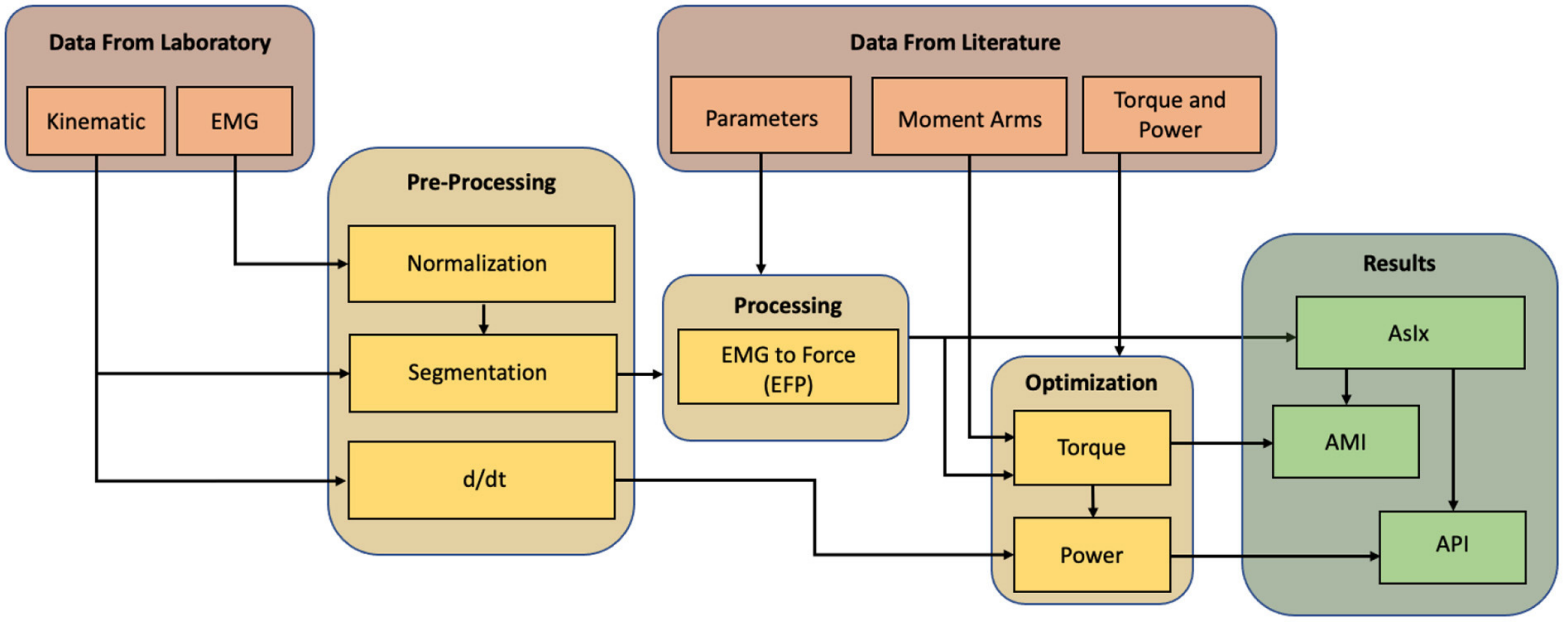
Methodology workflow. Data recorded in the laboratory is pre-preprocessed and integrated with data taken from literature to compute the EMG to force processing. The output is compared with the literature and optimized to approach the same trends. After the optimization, the assistive indexes can be calculated.

Figure 1 shows the fundamental steps in the analysis. Data from both the subjects under investigation and from the literature are used. Joint rotations and muscle activations are extracted from the former, while parametric data can be derived from the latter. After their acquisition, data from the laboratory must be pre-processed, segmented, and, if they are EMGs, normalized to be comparable with each other. Combining them with data from the literature it is possible to perform the EMG to Force Processing (*EFP*) and thus estimate the force delivered by each muscle while accomplishing the task. The obtained force signals are multiplied by the moment arms of their respective muscle tendons to calculate torques, and these are multiplied by the joint/limb rotation velocities (ω) to calculate the power at each joint. The torque and power signals are compared with those in the literature to find a good correlation coefficient. If the comparison is unsatisfactory, an optimization process of amplifying the force signals and re-calculating the kinetic parameters is computed. The force signals are also used to calculate each muscle's Assistive Index (*AsIx*), which are binary vectors representing the instants when muscle assistance is provided. Combining torque and AsIx signals makes it possible to obtain the Assistive Moment Indices (*AMI*), and therefore get an estimate of the assistance received in terms of joint moments. At the same time, by combining the power and the AsIx, it is possible to calculate the Assistive Power Indices (*API*) and assess the overall assistance in terms of joint power.

### 2.4. Pre-processing

**Figure 2:**
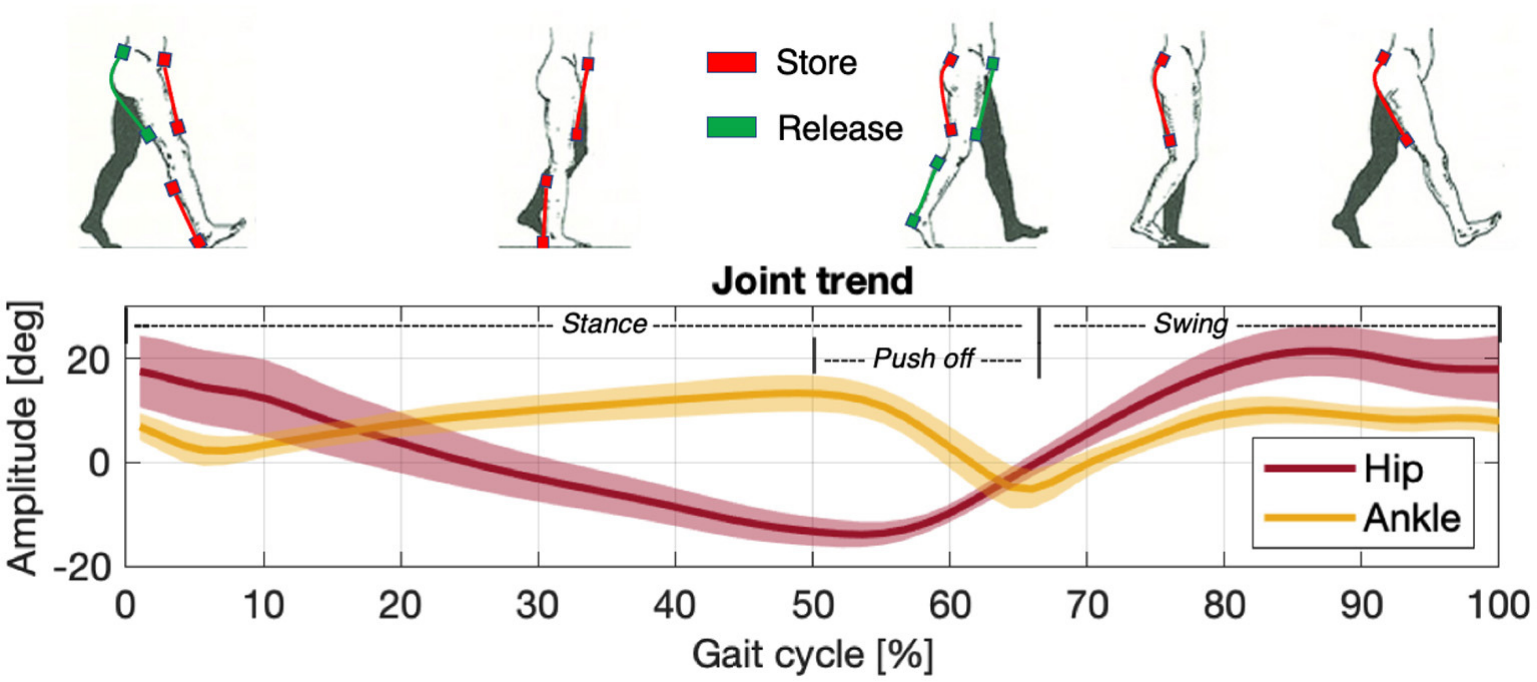
Storage (red) and release (green) phases of the actuation unit compared against the joint/limb rotation patterns of the hip (dark red) and ankle (yellow) in a gait cycle.

Before proceeding with EFP it is necessary to normalize and segment the EMG data (Burden et al., 2003; Sousa and Tavares, 2012). Normalization is usually done by dividing the signal by the maximum recorded voluntary contraction (MVC). The signals between different experimental trials must be comparable, and the proportions between the muscle's activation level and the force delivered must be maintained. To derive the EFP, the muscular signal envelopes are multiplied by their values of maximum isometric contraction (*F*_*max*_), which are available in the literature (Arnold et al., 2010) or may be calculated as reported in (9). The resulting signals express forces proportional to the muscle activations and are thus proportional to the force delivered by the users themselves. If no MVC has been recorded, it is also possible to normalize by dividing the signal by the maximum muscle activation expressed for the task (i.e., the peak activation level obtained during the task under investigation). In this case, however, the electromyographic signal is proportional to the muscle's overall force deliverable, not the force used to accomplish the specific task. Segmentation is a classification process to divide signals into portions with common characteristics that allow them to be considered stationary (Azami et al., 2011). Since walking is a repetitive event, the gait cycle is considered a portion of the signal equal in duration to the time between two consecutive heel strikes, called the stride (Winter, 2009; Whittle, 2014). Each stride comprises two sub-phases called stance (when the foot is in contact with the ground) and swing (when it is not). Between stance and swing, the ankle is responsible for the push-off (i.e., propelling the force to lift the leg). To carry on the analysis, both muscular and kinematic signals are segmented in gait cycles, Figure 2. Further information is shown in Section 3.2.

To calculate the joint power, it is necessary to multiply the joint torque by the corresponding joint velocity. The angular velocity of the joint (ω) can be easily calculated by taking the first derivative of the joint kinematic signal (θ).

### 2.5. EMG to force processing

**Figure 3:**
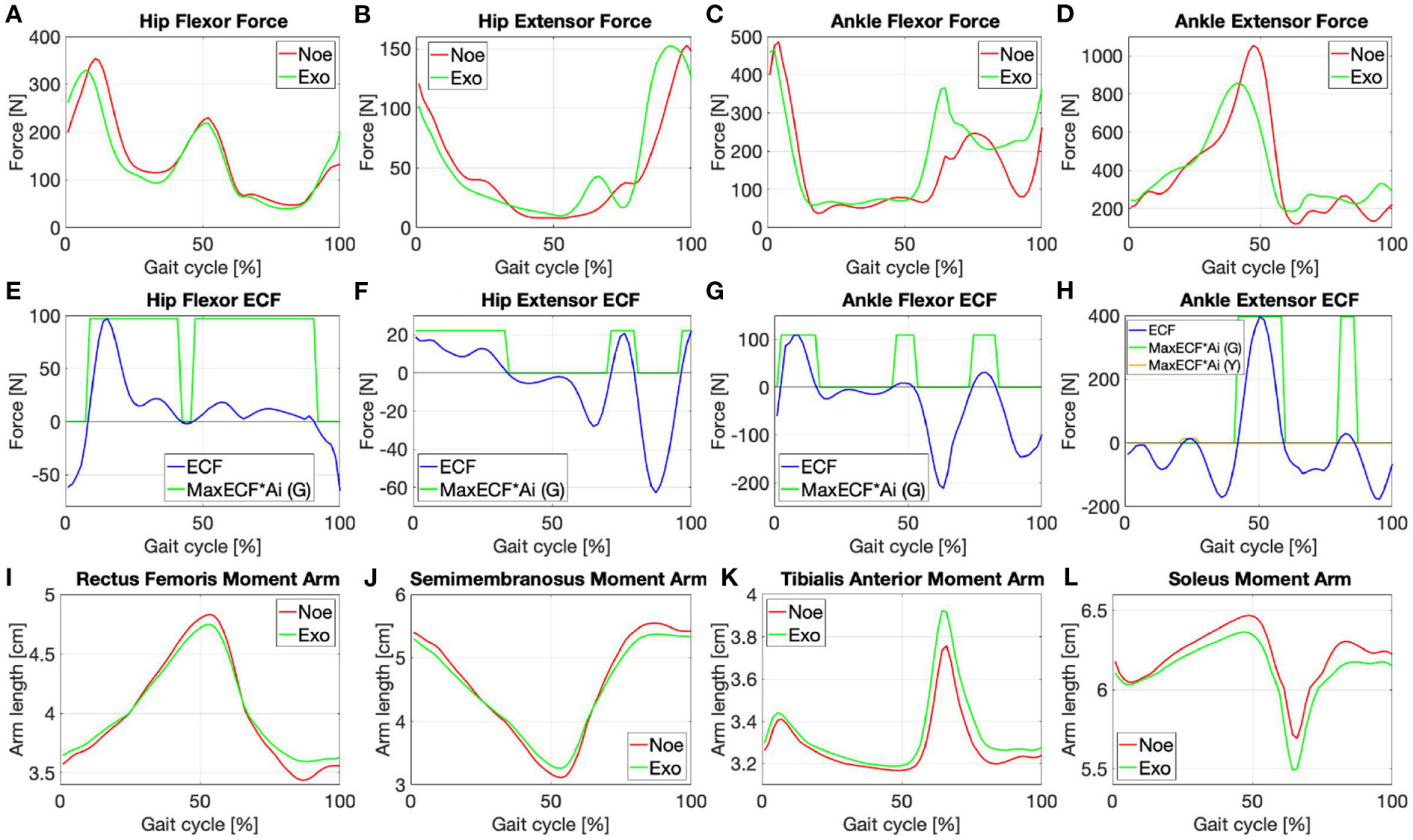
Force signals of the **(A)** rectus femoris, **(B)** semimembranosus, **(C)** tibialis anterior, **(D)** gestrocnemius medialis in the *Noe* (red) and *Exo* (green) configurations; the ECF of each muscle with the associated AsIx multiplied with a gain factor to be more easily viewable **(E–H)**; moment arms in the *Noe* (red) and *Exo* (green) configurations needed to calculate the torque generated by each muscle **(I–L)**.

With EMG to Force Processing it is possible to estimate the forces generated by the muscles under analysis, Figures 3A–D. It is based on the proportion between muscle activation and force production and relies on the Hill musculoskeletal model (Hof and Van den Berg, 1981; Bogey et al., 2005; Geyer and Herr, 2010). Following the steps below (Markowitz and Herr, 2016) the resultant of the forces acting on the muscle (*F*_*M*_) can be calculated as:


(1) $$\begin{array}{c} F_{M}\left(\alpha ,l_{CE},v_{CE}\right)=F_{CE}\left(\alpha ,l_{CE},v_{CE}\right)+F_{PE}\left(l_{CE}\right)-F_{BE}\left(l_{CE}\right); \end{array}$$

Where *F*_*CE*_ is the force produced by the contractile element (CE), *F*_*PE*_ is the force produced by the elastic structures surrounding the muscle (i.e., passive elements), and *F*_*BE*_ is the force produced by the buffer elasticity that prevents the muscle fascicle from shortening excessively.

According to the definition of *F*_*CE*_, the contractile component of *F*_*M*_ can be defined as:


(2) $$\begin{array}{l} F_{CE}=\alpha *F_{max}*fl\left(l_{CE}\right)*fv\left(v_{CE}\right); \end{array}$$

With α as the muscle activation (i.e., EMG signal filtered, rectified, normalized and segmented. See Section 3.5), *F*_*max*_ as the maximum isometric force, and *fl*(*l*_*CE*_) and *fv*(*v*_*CE*_) are respectively the active force-length and the force-velocity relations that are given by:


(3) $$\begin{array}{l} fl\left(l_{CE}\right)=\frac{-1}{w^{2}}*{\left(\frac{l_{CE}}{l_{opt}}\right)}^{2}+\frac{2}{w^{2}}*\frac{l_{CE}}{l_{opt}}-\frac{1}{w^{2}}+1; \end{array}$$

(4) $$fv(v_{CE})=\{\begin{array}{cc} N-\frac{\left(N-1)*(v_{max}-v_{CE}\right)}{7.56*K*v_{CE}+v_{max}} & \text{if }v_{CE}\geq 0;\\ \frac{v_{max}+v_{CE}}{v_{max}-K*v_{CE}} & \text{if }v_{CE}<0; \end{array}$$

Where *l*_*CE*_ is the contractile element length and *l*_*opt*_ is the CE length at which the muscle can provide the maximum force *F*_*max*_ (Eilenberg et al., 2010), *w* determines the width of the active force-length relation, *v*_*max*_ is the maximum muscle velocity, *K* is a curvature constant, and *N*, set as 1.5 (Markowitz and Herr, 2016), is the muscle force (in units of *F*_*max*_) at the muscle's maximum lengthening velocity.

The force arising from the elastic structures surrounding the muscle (*F*_*PE*_) and the force involved in the buffer elasticity (*F*_*BE*_) are defined as:


(5) $$F_{PE}(l_{CE})=\{\begin{array}{cc} F_{max}*{\left[\frac{\left(l_{CE}-l_{opt}\right)}{\left(l_{opt}*w\right)}\right]}^{2} & \text{if }l_{CE}\geq l_{opt};\\ 0 & \text{if }l_{CE}<l_{opt}; \end{array}$$

(6) $$F_{BE}(l_{CE})=\{\begin{array}{cc} F_{max}^{\frac{2}{w}}*{\left[\frac{\left(l_{CE}-l_{opt}*(1-w)\right)}{l_{opt}}\right]}^{2} & \text{if }\frac{l_{CE}}{l_{opt}}\leq (1-w);\\ 0 & \text{if }\frac{l_{CE}}{l_{opt}}>(1-w); \end{array}$$

With (*l*_*CE*_/*l*_*opt*_) intended as the strain of the fibers with respect to their optimal length (Arnold and Delp, 2011). Muscle fiber velocity *v*_*CE*_ is defined as:


(7) $$\begin{array}{l} v_{CE}=\overset{•}{l_{CE}}; \end{array}$$

where *l*_*CE*_ can be calculated as:


(8) $$\begin{array}{l} l_{CE}=\frac{l_{CE}}{l_{opt}}*l_{opt}; \end{array}$$

and *l*_*opt*_ is taken from Arnold et al. (2010). *F*_*max*_ can be calculated as:


(9) $$\begin{array}{l} F_{max}=PCSA*\text{Ψ}; \end{array}$$

Ψ is the maximum isometric stress value (Arnold et al., 2010), and the physiological cross-sectional area (PCSA) (Martin et al., 2020) is defined as:


(10) $$\begin{array}{l} PCSA=\frac{M_{M}*cos\left(\gamma \right)}{\rho *L_{f}}; \end{array}$$

Where *M*_*M*_ is the muscle mass, γ is the pennation angle of the muscle, ρ is the density of the muscle and *L*_*f*_ is the normalized muscle fiber length (Ward et al., 2009).

### 2.6. Optimization

**Figure 4:**
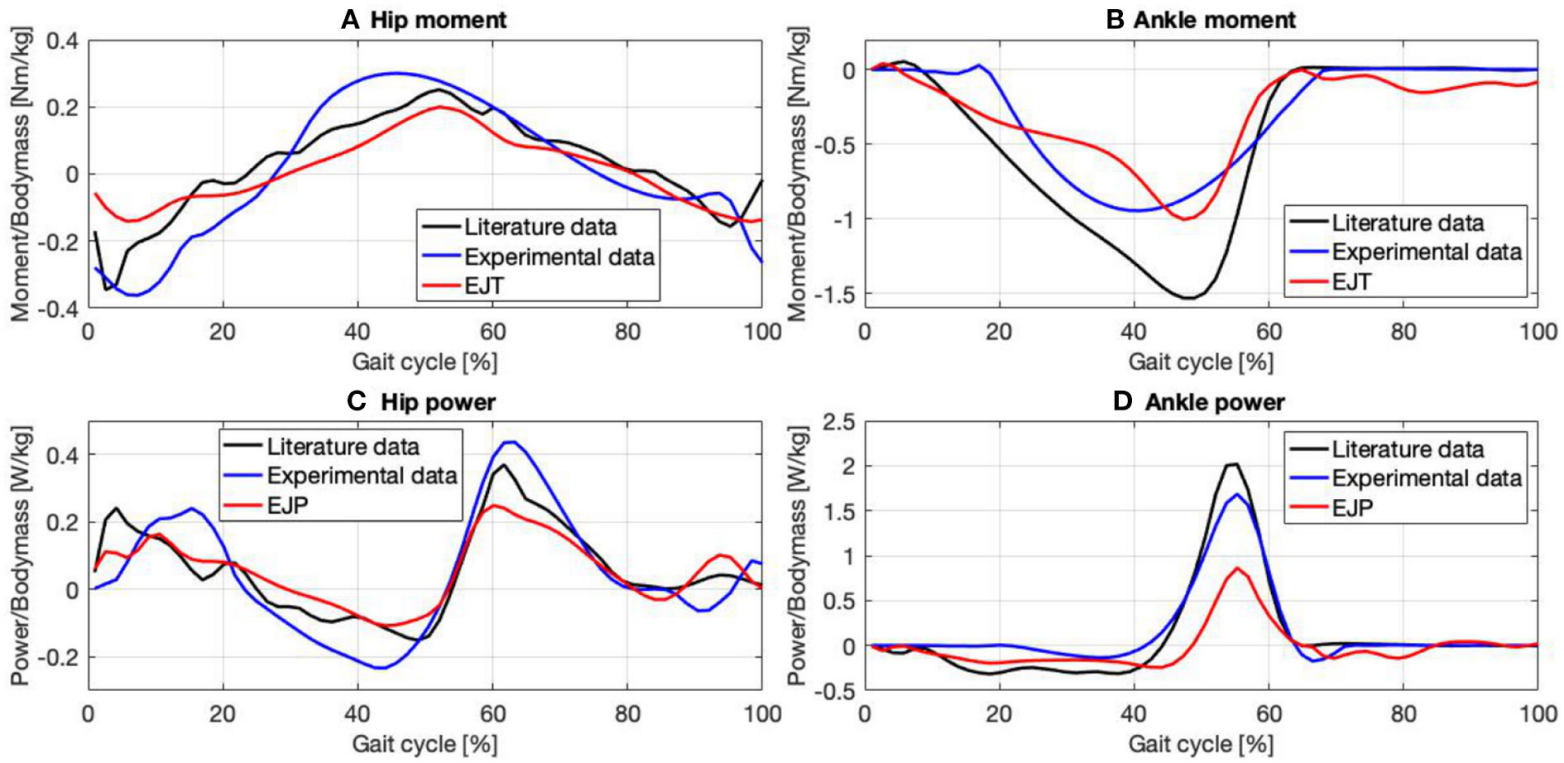
Joint moment and power for the hip **(A,C)** and the ankle **(B,D)** obtained with EMG to force processing (red trend) are compared with data gathered from literature (Winter, 2009) (black trend) and with experimental data obtained using force plates and inverse dynamic (blue trend).

The optimization process consists of calculating the joint torque and power signals and comparing them with data in the literature. It is needed to balance the opposing forces generated by the muscles that act on each joint. Each force signal contribution is modulated by multiplying it with a coefficient (*c*_1_, *c*_2_, etc.) that can vary according to the requirements. As reported in Winter (2009) “joint moments of force are the net result of all internal forces acting at the specific joint.” Therefore, the force delivered by the flexor and extensor muscles times their respective moment arms (*R*), Figures 3I–L, results in an estimation of the moments (*T*) acting on the specific joint, Figures 4A,B:


(11) $$\begin{array}{c} T_{Fl}=c_{1}*F_{Fl}*R_{Fl};\\ T_{Ex}=c_{2}*F_{Ex}*R_{Ex}; \end{array}$$

According to Spoor (1991) *R* is the first derivative of the instantaneous tendon length acting on the joint under analysis, as a function of the joint angle.

Considering the direction in which any muscle acts, to calculate the net of the moments acting on the joint, it is conventional to subtract the signal of the extensor moments from those of the flexor, to give:


(12) $$\begin{array}{c} T_{Noe}=c_{1}*T_{NoeFl}-c_{2}*T_{NoeEx};\\ T_{Exo}=c_{1}*T_{ExoFl}-c_{2}*T_{ExoEx}; \end{array}$$

Then, by multiplying the signals in (12) by the angular velocity of the joint (ω) (see Section 2.4), the mechanical power (*P*), Figures 4C,D, can be estimated as:


(13) $$\begin{array}{c} P_{Noe}=T_{Noe}*\omega_{Noe}=\left(c_{1}*T_{NoeFl}-c_{2}*T_{NoeEx}\right)*\omega_{Noe};\\ P_{Exo}=T_{Exo}*\omega_{Exo}=\left(c_{1}*T_{ExoFl}-c_{2}*T_{ExoEx}\right)*\omega_{Exo}; \end{array}$$

The obtained signals are optimized against literature gold standard signals (Winter, 2009), Figures 4A–D. The torque and power trends are calculated for each joint of interest (hips and ankles) and experimental modalities (with and without the exoskeleton) determining the signal of interest for the proposed analysis. We identify these trends as joint torque and joint power on the basis of EFP, namely: EMG to Joint Torque (EJT) and EMG to Joint Power (EJP). For what concerns the optimization we considered that for our purpose obtaining a Correlation Coefficient (CC), Fisher (1992), of 90% could be enough to show the user-exoskeleton interaction. The optimization process (Nelder and Mead, 1965), as shown in Equation (14), consists of a comparison of the torque and power calculated, to match the torque and power in the literature.


(14) $$\begin{array}{c} d_{1}=\overset{N}{\underset{i=1}{\sum }}|T_{Noe}\left(i\right)-T_{Lit}\left(i\right)|;\\ d_{2}=\overset{N}{\underset{i=1}{\sum }}|P_{Noe}\left(i\right)-P_{Lit}\left(i\right)|; \end{array}$$

*d*_1_ and *d*_2_ represents the distances between the functions, *N* is equal to the signal length, and *T*_*Lit*_ and *P*_*Lit*_ are respectively the torque and the power taken from Winter (2009).

Based on the result, the force signals are modulated in amplitude (acting on *c*_1_, *c*_2_, etc.) and the kinetic signals are calculated again until CC≥90%.

### 2.7. Kinetic parameters outcomes

To complete the analysis, it is necessary to calculate the Assistive Indexes. AsIx's are binary vectors specific for each muscle representing the instants of muscular assistance and are calculated by subtracting the *Exo* force signal from the *Noe* force signal (*F*_*Noe*_ − *F*_*Exo*_). The resulting signal is called below the Effort Comparison Function (ECF), Figures 3E–H. AsIx's signals are calculated for both the flexor and the extensor muscles of every joint (Equation 16) and are represented as 1 when the muscle is receiving assistance (i.e., ECF≥0), and 0 while the muscle is performing more effort compared to the baseline condition (i.e., ECF < 0):


(15) $$AsIx_{Fl/Ex}=\{\begin{array}{cc} 1 & \text{if }(F_{Noe}-F_{Exo})\geq 0;\\ 0 & \text{if }(F_{Noe}-F_{Exo})<0; \end{array}$$

During the AsIx calculation, the ECF of each muscle is divided by its respective force signal in the *Noe* configuration. If the result is ≥0.1 the assistance received is greater than 10% of the force delivered by the muscle at that instant, and the index (AsIx (G)) is green; if the result is <0.1 the assistance is present but very low, and the index (AsIx (Y)) is yellow. The signals for the flexors and extensors are logically joined when they are positive, to have a single signal for each color that refers to the whole joint:


(16) $$\begin{array}{c} AsIx\left(G\right)=AsIx_{Fl}\left(G\right)\cup AsIx_{Ex}\left(G\right);\\ AsIx\left(Y\right)=AsIx_{Fl}\left(Y\right)\cup AsIx_{Ex}\left(Y\right); \end{array}$$

Moreover, a No-AsIx signal is computed to take into account the instants when neither AsIx (G) nor AsIx (Y) are positive:


(17) $$\begin{array}{c} No-AsIx=\neg \left(AsIx\left(G\right)\cup AsIx\left(Y\right)\right); \end{array}$$

It must be noted that when referring to AsIx while calculating kinetic parameters, the same procedure has to be applied to both the AsIx (G), the AsIx (Y) and the No-AsIx signals.

Once the AsIxs have been computed it is possible to estimate the Assistive Moment Trend (AMT), Figures 4A,B, as:


(18) $$\begin{array}{c} AMT=T_{Noe}-T_{Exo}=\\ =\left(F_{NoeFl}*AsIx_{Fl}*R_{NoeFl}-F_{NoeEx}*AsIx_{Ex}*R_{NoeEx}\right)-\\ \left(F_{ExoFl}*AsIx_{Fl}*R_{ExoFl}-F_{ExoEx}*AsIx_{Ex}*R_{ExoEx}\right); \end{array}$$

and the Assistive Power Trend (APT), Figures 4C,D, can therefore be calculated as:


(19) $$\begin{array}{l} APT=P_{Noe}-P_{Exo}\\ =T_{Noe}*\omega_{Noe}-T_{Exo}*\omega_{Exo}\\ =\left[\left(F_{NoeFl}*AsIx_{Fl}*R_{NoeFl}-F_{NoeEx}*AsIx_{Ex}*R_{NoeEx}\right)*\omega_{Noe}\right]\\ -\left[\left(F_{ExoFl}*AsIx_{Fl}*R_{ExoFl}-F_{ExoEx}*AsIx_{Ex}*R_{ExoEx}\right)*\omega_{Exo}\right]; \end{array}$$

The indices of assistance for the moments (AMI) and the powers (API) are calculated, in each interval (*K*) identified by the AsIx, as:


(20) $$\begin{array}{l} AMI=SignM_{\left(K\right)}*\frac{\left|\sum_{i=1}^{K}\left(T_{Noe}(i)-T_{Exo}(i)\right)\right|}{\sum_{i=1}^{K}\left|T_{Noe}(i)\right|};\\ API=SignP_{\left(K\right)}*\frac{\left|\sum_{i=1}^{K}\left(P_{Noe}(i)-P_{Exo}(i)\right)\right|}{\sum_{i=1}^{K}\left|P_{Noe}(i)\right|}; \end{array}$$

Where *SignM* and *SignP* represent a reduction (positive sign) or an increase (negative sign) of moment and power and are calculated in each interval (*K*):


(21) $$\begin{array}{c} SignM_{\left(K\right)}=\overset{K}{\underset{i=1}{\sum }}\frac{\left(T_{Noe}\left(i\right)-T_{Exo}\left(i\right)\right)}{T_{Noe}\left(i\right)};\\ SignP_{\left(K\right)}=\overset{K}{\underset{i=1}{\sum }}\frac{\left(P_{Noe}\left(i\right)-P_{Exo}\left(i\right)\right)}{P_{Noe}\left(i\right)}; \end{array}$$

## 3. Experimental validation

The proposed method has been applied and validated on a quasi-passive exoskeleton but could be extended to active and passive systems and this will form part of future work. Tests were performed in accordance with the experimental protocol approved by the Ethics Committee of Liguria, Italy (protocol number: 001/2019) and complied with the Helsinki Declaration.

### 3.1. XoSoft-Gamma

**Figure 5:**
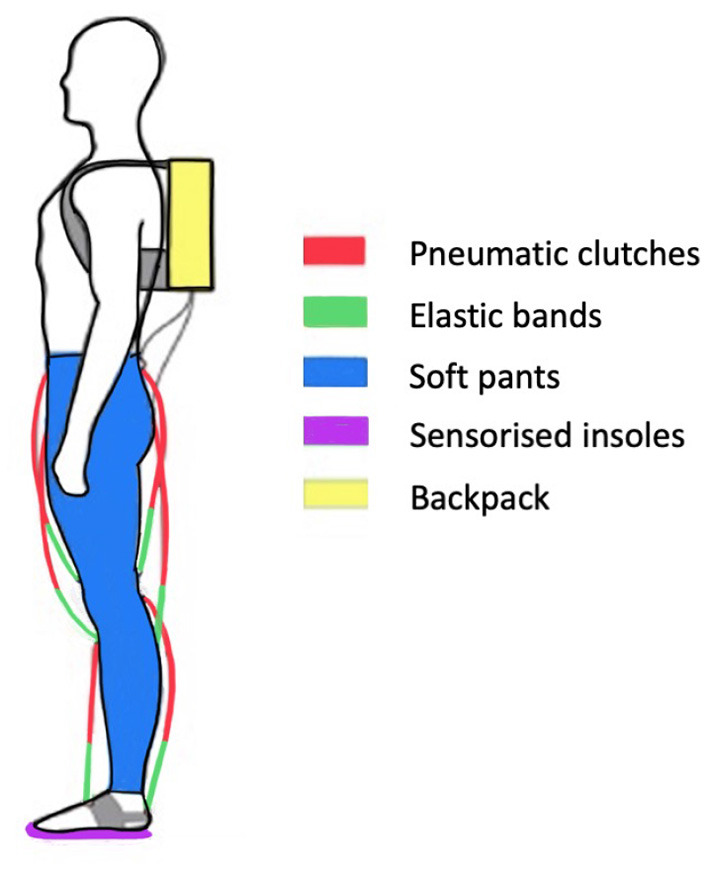
Structure of the XoSoft-Gamma exoskeleton. The exosuit consists of pneumatic clutches connected in series with elastic bands, soft pants, sensorized insoles, and a backpack containing electronic and pneumatic systems.

The presented methodology is evaluated on the soft exoskeleton platform XoSoft (Gamma version). XoSoft-Gamma is a lower limb exosuit developed within the XoSoft European project (Horizon, 2020) to assist human walking (Poliero et al., 2018). The assistance provided relies on quasi-passive actuators (Di Natali et al., 2019, 2020) developed to support/enhance mobility in people with lower-limb impairments (Power et al., 2016; Graf et al., 2018). The exosuit has a modular structure (Ortiz et al., 2019) with several arrangement configurations and can assist different joints at specific moments in the gait (Ortiz et al., 2018). As reported in Figure 5, it comprises soft pants, a backpack containing electronic and pneumatic systems, two sensorized insoles for step-phase identification (Mateos et al., 2016), and up to six biomimetic quasi-passive actuation units to assist an equivalent number of unidirectional movements. The actuation unit is a pneumatic soft clutch connected in series to passive elements. The clutch is used to modulate and control the elastic energy contribution with characteristics presented in Sadeghi et al. (2019a,b). The key feature of passive exoskeletons is the use of elastic and spring components that form conservative elements able to transduce kinetic energy in potential energy and vice versa. The clutch activation and deactivation phases are regulated by the control strategy, which also determines the amount of energy stored and released. In fact, depending on the characteristics of the elastic component (i.e., width, thickness, stiffness, and deformation), it is possible to quantify the energy contribution in absolute terms (Di Natali et al., 2019). By choosing the de/activation times of the clutch, it is possible to select the moments during the gait cycle when the energy is accumulated from the user or transmitted back (Di Natali et al., 2020).

### 3.2. Assistive and control strategy

The XoSoft actuators can assist the user's lower limbs in the sagittal plane. Each hip, knee, and ankle joint can be supported during flexion and/or extension (Di Natali et al., 2020). Thanks to the modularity of the exosuit, it is possible to assist a total of six joint/limb movements at different moments in the gait cycle. During the gait (Winter, 2009), the hip joint is responsible for moving the legs forward and, as a consequence, the whole body. It extends for the 60% of the gait cycle (stance) and flexes for the remaining 40% of the gait cycle (swing). The knee, starting from the standing position, only flexes and then moves the lower leg forward without hindering the movement. At the same time, the ankle plantarflexes between the 50 and the 70% of the gait cycle, transmitting the propulsive force necessary to initiate the swing phase (Wiggin et al., 2011). Subsequently, it dorsiflexes slightly before the next stride to prepare for the ground contact.

Based on our considerations and the literature (Shi et al., 2019), the movements that require a higher energy supply, according to the dynamics of the motion and the mass that needs to be propelled to accomplish each gait phase, are hip flexion, hip extension, and ankle plantar-flexion. We decided to assist these exact movements, and noted that several literature studies had focused on the same actions (Wiggin et al., 2011; Asbeck et al., 2013; Van Dijk and Van Der Kooij, 2014; Liu et al., 2019; Panizzolo et al., 2021). An important factor that has to be tuned for the actuators is the control timing of the clutches' engagement and disengagement. The criteria for the time selection include: Joint/limb rotation maxima and minima (i.e., gait phases during which joint biomechanics allows a passive actuation); Joint torque and power (i.e., the preferred range in which to receive assistance according to the joint torque and power); COM kinetic and potential energy (i.e., the preferred moment to actuate the hip-joint according to the COM energy, by approximating it on the pelvic zone) (Bennett et al., 2005); If testing is with non-healthy subjects there are any specific diseases/conditions that need special consideration.

Maxima and minima joint rotations and joint power were considered for both the hip and the ankle actuation time. Previous studies showed that with a quasi-passive actuation based on the joint rotation and the elastic elements' coefficients, it is possible to define a control strategy considering the storing and releasing time (Di Natali et al., 2020). For the hip has also been considered what reported in Bennett et al. (2005), and therefore to not start storing the energy in the actuator while the joint kinetic and potential energy are respectively in a local minimum and maximum (i.e., not transfer the energy between the user and the actuation unit in the 20, 75, and 95% of the gait). Finally, as all participants were healthy, no specific disease consideration was made.

According to what was exposed above the time of actuation chosen for assisting the hip flexion and extension and ankle during the push-off phases, Figure 2, are:

- Hip Flexion: 5% gait cycle - 65% gait cycle;
- Hip Extension: 50% gait cycle - 15% gait (next cycle);
- Ankle Plantar-flexion: 5% gait cycle - 60% gait cycle;

### 3.3. Experimental protocol

To investigate the method's efficacy the test was undertaken with 7 subjects, (5 men and 2 women), average age 29 ± 4 years, weight 65 ± 8Kg, height 175 ± 4cm. Data were initially acquired during 10 min of level-walking on a treadmill at a speed of 4 km/h (1,11 m/s) in the *Exo* mode, and then during 10 min of walking at the same speed in the *Noe* configuration. Between tests, the participants had 10 min of rest during which the backpack and the actuation systems were removed. The choice of the duration was made to acquire a substantial amount of data for each task, around 900 strides per subject, while the choice of analyzing first the walk with the exoskeleton and then without it was made to avoid the logistic problems that would have occurred trying to wear the exoskeleton after having already positioned the sensors and performed the first recording. To record the movement, an Xsens wearable motion tracking system was used (MTw Awinda 3D Wireless Motion Tracker, Xsens Technologies B.V. Enschede, the Netherlands) at a sampling rate of 60 Hz. The acquisition involved the use of seven motion trackers positioned on the feet, lower legs, upper legs, and pelvis. For muscle activity, an 8-channel Wi-Fi transmission surface electromyograph (FreeEMG300 System, BTS, Milan, Italy) was used at a sampling rate of 1,000 Hz. To ensure the correct acquisition of the signals and avoid displacements, the sEMG electrodes were positioned at a distance of two centimeters between each other, following the direction of the muscle fibers according to the European recommendations for surface electromyography (Hermens et al., 2000). Moreover, the sensors were attached to the body through elastic straps that held them firmly against the skin.

For what concerns muscular fatigue, a test designed to walk on a treadmill for 10 min does not generate a substantial difference in the signal. Muscular fatigue affects the mean frequency as demonstrated in a specific work where a resistive exoskeleton was controlled with the aim to fatigue muscles (Di Natali et al., 2021). In Ament et al. (1993) has been shown a reduction of the mean frequency of 7.5% (5% for the soleus muscle and 10% for the gastrocnemius medialis) obtained after 10 min running activity. Since the goal of this work is not to monitor the mean frequency index, the experimental protocol should not create any modification of the muscular signal readings.

### 3.4. Muscle contribution and EMG assessment

The EMG sensor positioning is not always easy when testing an exoskeleton. The structure of the exoskeleton could obstruct access to some body areas preventing sensor placement, and the actuation system could rub off the EMGs displacing or removing them. To overcome these problems preliminary tests have been performed to select the most suitable/responsive muscles for flexion and extension movements during walking and the most easily accessible signals recorded with the sEMG. Friction and the encumbrance of the actuators pressing on the sensors have also been considered. As a result, we chose to record the: rectus femoris (hip flexor), semitendinosus (hip extensor), tibialis anterior (ankle dorsi-flexor), and gastrocnemius lateralis (ankle plantar-flexor).

Two considerations were made: (i) some muscle bundles are part of the same muscle group and although they have different force levels they have very similar electrical envelopes; (ii) some joints such as the hip are driven by multiple muscles. Considering that joint moment and power are derived from the co-activation of all of these, it may be necessary to assess the contribution of more flexor/extensor muscles than recorded.

**Figure 6:**
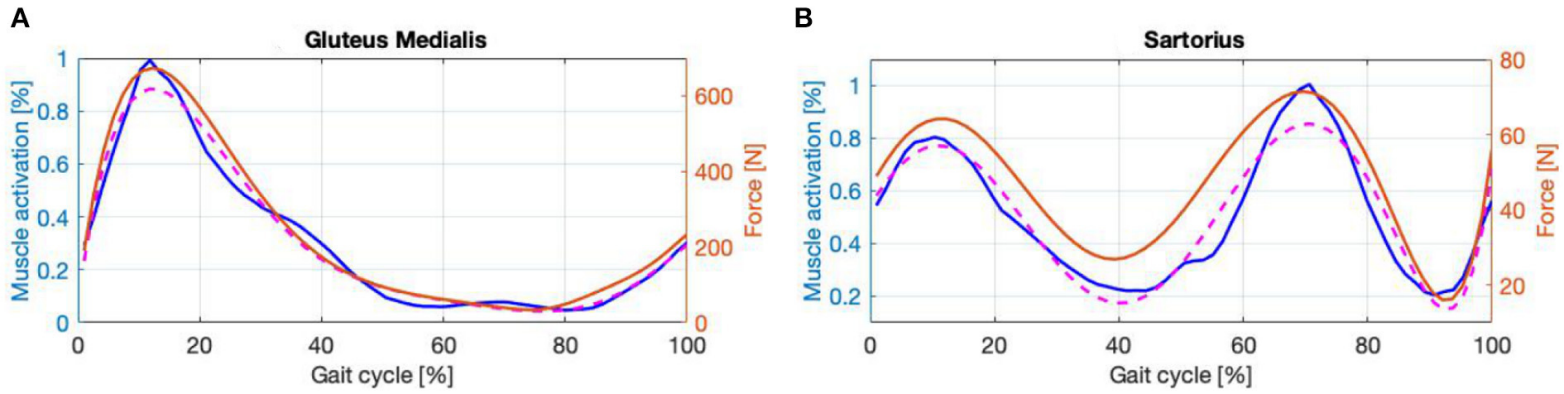
**(A,B)** Envelope (blue line), interpolatin curve (dashed line) and force signal (magenta line).

As an example of (i), the calf comprises two external muscle bundles that divide it into medial and lateral gastrocnemius, plus an inner muscle bundle named the soleus. The soleus can generate more force than the gastrocnemius, but it is more difficult to observe because of its position. On the other hand, the lateral gastrocnemius creates less force but is easier to reach. For these reasons and due to the similarity of their envelopes, as our aim is to assess the overall forces acting on the joint, it was decided to record the activity of the lateral gastrocnemius but attribute to it the characteristics of the strongest muscle of the group during EFP (i.e., the soleus). The same reasoning was made for the thigh extensors by recording the activity of the semitendinosus, but attributing to it the characteristics of the semimembranosus. For assumption (ii), the hip extensors are divided into two main muscle groups which are the hamstring and the gluteus. As reported in Winter (2009) the gluteus and hamstring have very different envelopes but work closely together in hip extension. Due to the encumbrance of the exoskeleton, it was difficult to monitor the muscular activity of the gluteus. Thus, the envelope of the gluteal EMG signal, extracted from Winter (2009), was interpolated with a 6th-order curve showing a correlation coefficient (CC) of 99%. The features of the gluteus medialis were assigned to this signal during the EFP, Figure 6A, and the derived force was considered as a contribution to the hip extension. The same reasoning was followed for the hip flexion with the sartorius, Figure 6B, showing a CC of 96.35%.

### 3.5. Data analysis and model setting

Data were processed using MATLAB software (MATLAB 9.7.0, MathWorks, Natick, MA, USA). The recorded EMG data were filtered using a fourth-order Butterworth filter with a bandwidth between 30 and 450 Hz. This was rectified and treated again with a Butterworth second-order low-pass filter with a cut-off frequency of 10 Hz. The EMG signals were normalized with the maximum activation of each muscle in the *Noe* condition. Each signal was therefore proportional to the maximum force exerted by the respective muscle during walking without aid from the exoskeleton. The resulting signals were segmented into gait cycles.

For force processing the (*l*_*ce*_/*l*_*opt*_) relationship was derived from Arnold and Delp (2011) for all the muscles, while *F*_*max*_ was calculated as shown in Equation (9). In particular PCSA values have been obtained from Handsfield et al. (2014) and the maximum isometric stress value (Ψ) has been set to 41.75 [N/cm^2^] (i.e., average of the values found in literature Arnold et al., 2010). The trend of *R* as function of the joint angulation is estimated for the rectus femoris and gluteus medialis (Zheng et al., 2008), for the semimembranosus (Arnold et al., 2000), for the tibialis anterior (Maganaris et al., 1999), for the soleus (McCullough et al., 2011) and for the sartorius (Scheys et al., 2008). For the sartorius, as an approximation, the relationship between the moment arm length and joint angle has been considered linear. With the obtained values, it was possible to interpolate the curves representing the moment arms of each muscle in a gait cycle, as shown in Figures 3I–L. The parameters for the EFP were taken from Geyer and Herr (2010) and its Supplementary material for all muscles except the sartorius, which was not included. For the sartorius *v*_*max*_ and *K* were calculated as in Geyer and Herr (2010) considering the fraction of fast-twitch fiber (FFT) in the muscle to be equal to 1 (Close and Hoh, 1968), and *w* as for the rectus femoris. Finally, when calculating the AsIx, we highlight in green (G) the contours of the phases during which the assistance received (in terms of [N]) is greater than 10% of the muscular force at that instant; values below 10% were highlighted in yellow (Y).

## 4. Methodology validation

### 4.1. Kinetic parameters validation

To validate the results, moments and powers found in literature (Winter, 2009) were compared against experimentally obtained EJT and EJP results from the proposed methodology. Data for the hips and ankles are shown in Figures 4A–D, with cross-referencing correlation coefficients reported in Table 1. Thanks to optimization, where the muscles' forces are combined to generate torque and power, amplification coefficients can be selected to properly modulate the contribution of each muscle group, Table 2. The technique described in this paper can use either data gathered from literature or available database. We have selected data from Winter (2009), which have been calculated from inverse dynamics. This dataset has been collected on 60 subjects, thus providing a solid dataset that can be used as the moment and power reference baseline. Moreover, this dataset is also anthropometrically similar to the experimental sample of this study (Winter: number = 60 healthy subjects; Age = (20–49) years; Height = 176 cm; Weight = 72 kg. Our study: number = 7 healthy subjects; Age = 29 years; Height = 175 cm; Weight = 65 kg). The results in the figures and tables represent the average trends and values.

**Table 1:** Correlation coefficient of the hip and ankle moments and powers.

**Signal**	**Proposed methodology**	**Experimental data**	**Proposed methodology**
	**vs**.	**vs**.	**vs**.
	**Literature data**	**Literature data**	**Experimental data**
	**(CC-ML)**	**(CC-EL)**	**(CC-ME)**
Hip moment	90.1%	93%	90.9%
Ankle moment	94.8%	95.1%	88.6%
Hip power	90.8%	89.7%	91.9%
Ankle power	90.4%	97.6%	90.3%

CC-ML is the CC obtained by comparing data from the literature with the EJT and EJP. The CC-EL is obtained by comparing the inverse dynamics from the experimental data against results from the literature (Winter, 2009). CC-ME is obtained by comparing the EJT and EJP against the signals calculated by inverse dynamics.

**Table 2:** Amplification coefficients for the joint movements and their associated muscles.

**Joint movement**	**Muscle bundle**	**Amplification coefficient**
Hip flexion	Rectus femoris	0.2
Hip extension	Semitendinosus	0.1
Hip flexion	Sartorius	0.55
Hip extension	Gluteus medialis	0.4
Ankle flexion	Tibialis anterior	0.65
Ankle extension	Gastrocnemius lateralis	0.15

All three hip moment profiles, Figure 4A, have similar trends, with correlation coefficients (CC) always >90%, Table 1. The plots from the literature dataset, (black), show similar trends and amplitudes to those obtained by applying inverse kinematics on the experimental data, (blue), and the plot calculated from the EFP (red). The relative errors (RE) between functions, calculated over the entire gait cycle and normalized to the maximum of the reference signal using literature data when possible, and experimental data otherwise are: RE-ML=6.1 ± 18.2%, RE-EL=6 ± 25.4%, and RE-ME=0.1 ± 32.5%. The main differences occur at the start of the gait cycle, where the amplitudes are very heterogeneous, and between 30 and 50% of the gait cycle, where the experimental result diverges slightly from the other plots. The relative error associated with the signals in the 30–50% interval are: RE-ML=17.7 ± 3.8%, RE-EL=26.9 ± 8.9%, and RE-ME=42.6 ± 10.4%. The inflection points are consistent except in the final phase of the cycle. Between 90 and 100%, in fact, the experimental plot follows a negative slope, while the EJT and literature trends have a positive slope. The ankle moment, Figure 4B, as shown in Table 1, has correlations close to 95% for both CC-ML and CC-EL, with CC-ME having a slightly lower correlation value, 88.6%. Despite amplitude differences in the mid-cycle, the literature and EJT plots have very similar trends and the same inflection points. The trend for the experimental data is similar to both the other plots with amplitude comparable to the EJT but the inflection point is 10% earlier in the cycle. Although the amplitude of the experimental data and the EJT are comparable, the relative errors between the signals are RE-ML=10.22 ± 18.3%, RE-EL=10.17 ± 15.5%, and RE-ME=0.1 ± 18.5%. During the swing phase, the experimental and literature data show a trend close to 0 [Nm/kg], while the EJT shows some activity, which results in a slight negative trend. Power at the hip, Figure 4C, shows very similar trends for all the signals. At a detailed level there are of course differences, with the most noticeable being phase advances and delays in the experimental plot, particularly at 15, 40, and 100% of the gait cycle. The three relative errors are: RE-ML=0.1 ± 13.3%, RE-EL=2.2 ± 22.8%, and RE-ME=1.7 ± 22.4%. The ankle power, Figure 4D, has the highest correlation level for plots obtained from inverse kinetics, 97.6%, with values above 90% for the other two cases. The relative errors are: RE-LM=6.2 ± 17.2%, RE-EL=2.4 ± 7.6% and RE-ME=10.2 ± 15.2%, but between 40 and 65% this error increases to: RE-ML=27.3 ± 22.9%, RE-EL=2.3 ± 9.7% and RE-ME=29.9 ± 18.4%. As with the ankle moment, there is some EJP activity during the swing phase.

### 4.2. Kinetic parameters discussion

It should be stressed that the methodology presented in this paper does not aim for a gold-standard levels of accuracy, rather it is intended for, and can provide, real-time joint moments and powers over an extended time period. Hence it can form a tool in a user-centered design approach that can be used in the iterative development of exoskeletons and for assistive control strategy optimization. Note that: the number of muscular signals selected to reproduce the antagonistic force aimed to achieve a correlation >90% with literature data and over 85% in the other cases.

Although the hip is a complex joint, four signals were enough to reproduce its moment and power, Figures 4A,C, with a good approximation. For the hip and ankle moments, Figures 4A,B the CCs when compared with data from literature are always >90% apart from the ankle moment where CC-ME is still a very good 88.6%. For the EJT signal, Figure 4A, both the first and the last negative peaks are mainly due to the contribution of the hip extensor recorded in the lab. Therefore, it was necessary to find a median value for both the first and the last part of the cycle. Between 20 and 50%, the EJT has a constant deviation with respect to literature data due to the hip flexor recorded in the lab and the hip extensor gathered from the literature. The hip flexor recorded in the lab is also responsible for the prominent peak at 50% of the cycle, while the hip flexor data from the literature is responsible for the descending slope at 60–80%. For the ankle moment, Figure 4B, only two muscles were required to provide 95% correlation. For the EJT signal, the small positive peak between 0 and 10% of the gait cycle is mainly due to the flexor muscle, while the following negative peak is due to the extensor. During the descent phase, the amplitude difference between the EJT and the literature-derived signal is notable, but the trends are similar and the inflection points between 30 and 40% are respected. The upward slopes between 50 and 60% of the cycle are both similar, although there is some difference in the subsequent swing phase. Unlike the experimental and literature plots, the EJT signal between 60 and 100% deviates from 0 [Nm/kg], due to the muscle activity seen in Figures 3C,D at the same point in the cycle. Considering the power plots for both the hips and ankles, Figures 4C,D, very similar trends are observed, with CCs above 90% except for the hip powers obtained by inverse dynamics, where CC-EL equals 89.7%. This is due to the phase shift in the experimental signal's first, second, and fourth peaks. Comparing the hip power EJP signal with the literature data, Figure 4C, the inflection points between 0 and 20% are respected. Also, as for the hip moment, the first peak is considerably reduced and the gap between the signals in the range 10–40% of the gait cycle is present. The subsequent upward and downward phases are very similar, especially considering trends and turning points. In the final phase, 90–100%, the difference between the signals, which was just noticeable in the hip moment, is now increased and results in a high positive peak. Finally, the ankle power, Figure 4D, has a flat, negative, and constant trend for the whole stance phase. The signals are particularly close until 40% of the cycle, then the divergence increases. During push-off, the trends are similar for both the ascending and descending phases, but, comparing the EJP and the literature data curves, the amplitudes differ. Again, considering more flexor muscle signals would increase convergence. During the swing phase, the effects are similar to those observed for the ankle. The values for the literature and experimental curves approach 0 [W/kg], but the EJP plot shows some muscle activity.

The plots in Figures 4A–D, and the high correlation between the experimental and literature data provide high confidence that the literature data can be used as a baseline for calibration of the kinetic signals calculated *via* EFP. Since the EJT and EJP signals using the Noe configuration have a high correlation with the signals from both literature and inverse dynamics we believe that the method developed here can successfully generate kinetic trends from EMG signals. Subsequently, the amplification coefficients, Table 2, can be used to calculate the signals in the Exo configuration and evaluate the exoskeleton performance.

## 5. Exoskeleton assessment: Results and discussion

As a part of the discussion could be relevant to consider a comparison of the proposed method against the kinetic analysis performed with the inverse dynamics method as done in Di Natali et al. (2020) on the same exoskeleton platform. The traditional analysis that focuses on extracting single indices (EMG, torque, power, etc.) describes the complex problem in a partial way. Despite the different control strategies, in fact, in Di Natali et al. (2020) it can be seen that what is usually considered assistance does not necessarily correspond to a positive contribution. Indeed the selected indices are partial and do not represent the complex scenario. With these considerations in mind, we take the cue to evaluate the performance of the exoskeleton using the proposed and validated analysis. Moreover, considering that the exoskeleton platform used to validate this method is XoSoft, i.e., a quasi-passive exoskeleton used to assist the walking, its use aims to reduce and re-modulate the user's kinetic contribution during tasks such as walking (see Section 2.1). The results of the assistance are reported in Table 3.

**Table 3:** Gait cycles of the hip and the ankle with their percentage breakdowns.

**Gait cycle %**	**Color**	**AMI [Nm/kg]**	**API [W/kg]**
**HIP**			
0–42%	G	–2.46	–0.41
42–47%	R	+0.21	+0.72
47–91%	G	+8.63	+13.07
91–96%	R	–0.57	+3.67
96–100%	G	+1.98	+0.99
**ANKLE**			
0–16%	G	–3.34	–1.97
16–21%	R	–0.40	–0.69
21–27%	Y	+0.35	+0.90
27–42%	R	–4.36	–0.74
42–60%	G	+14.59	+9.40
60–73%	R	+0.71	+9.81
73–86%	G	–0.47	–3.11
86–100%	R	–1.99	+1.47

The color of the area in the range, and the corresponding AMI and API are reported too.

**Figure 7:**
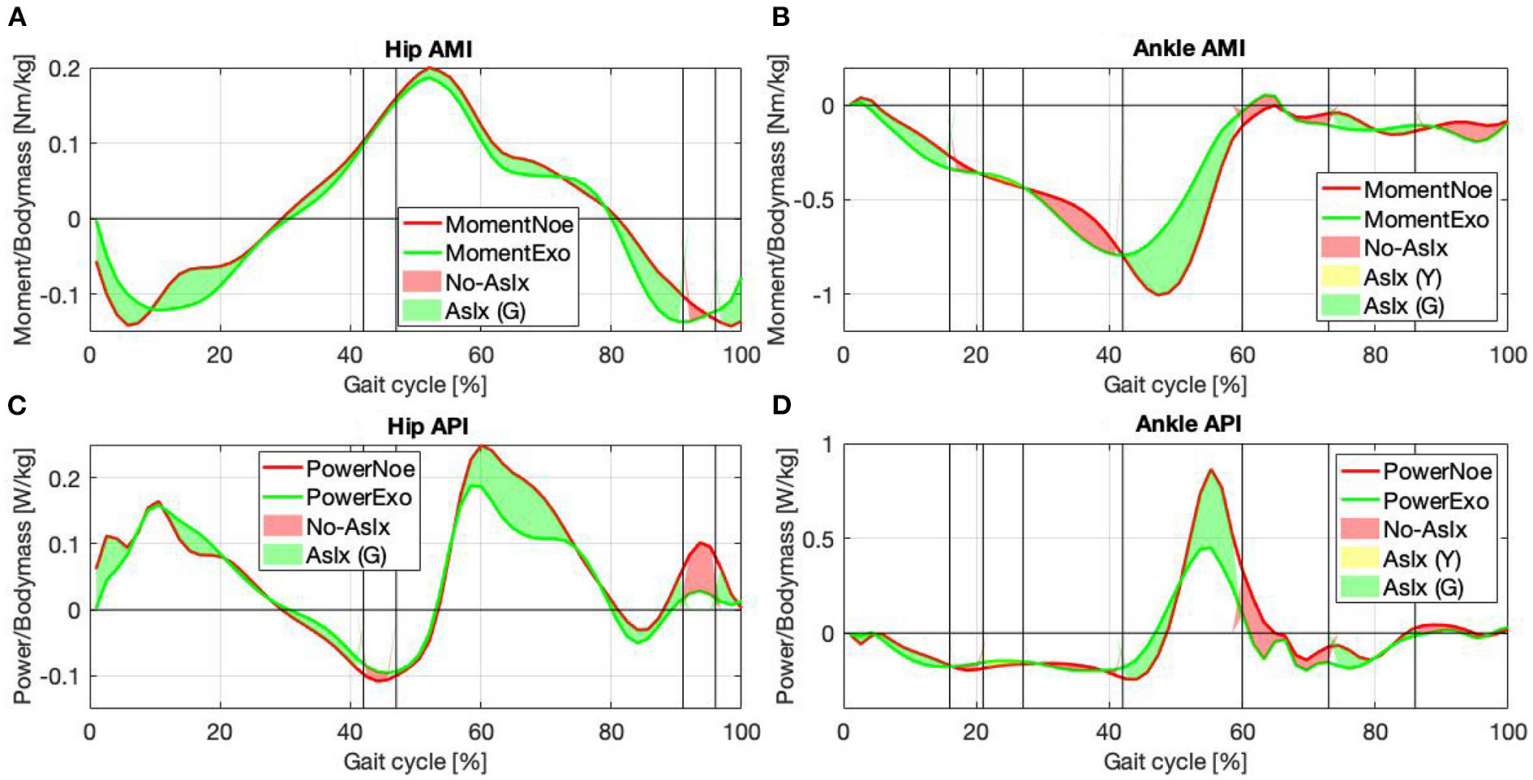
Joint moment and power for the hip **(A,C)** and the ankle **(B,D)** in the *Noe* (red line) and *Exo* (green line) configuration are compared. Areas represent the instants of major (green), minor (yellow), and null (red) assistance.

For the hip joint, Figures 7A,B, the values underline the effectiveness of the control strategy designed to assist the hip flexion and extension by re-modulating the user's energetic contribution. The gait phases during which at least one of the two muscles is assisted, green and yellow together, represent 90% of the whole gait cycle, of which half during the stance and half during the swing. Therefore, the hip is almost always assisted. The hip moment, Figure 7A, reports a gain of 8.15% [Nm/kg] when the color is green and a loss of 0.36% [Nm/kg] when it is red, suggesting that a considerable gain is associated with a very little loss. The most noticeable difference between the *Noe* and the *Exo* signals is between 47 and 91% of the gait cycle, where the color is green and the associated value is 8.63% [Nm/kg]. The interval represents a reduced torque requirement for the user while the muscles reduce their activity. The assistance is both kinetic and muscular (see Section 2.1). Moreover, adding positive values with each other and negative as well, the overall gain is 10.82% [Nm/kg], and the overall loss is 3.03% [Nm/kg], indicating that the torque released by the implementation is greater than that required to store energy in the system. Furthermore, if we consider that the subjects performed the test walking on a treadmill at a constant speed, any change of torque and power represents a variation in muscle pattern or a reduction of required force while performing the same cyclic motion. For hip power, Figure 7C, the gain is 13.65% [W/kg] when the color is green and 4.39% [W/kg] when the color is red. Therefore, the joint power is reduced both while the muscles are receiving assistance and when they are not. The only negative value is reported in the first part of the gait cycle, while the other phases are always associated with positive indices. The maximum gains are between 47–91% and 91–96% of the gait cycle, with corresponding values of 13.07%[W/kg] and 3.67%[W/kg]. Also in this case, the reduction of power is associated with both the stance and the swing phases. The overall power reduction, without distinguishing the different colors, is 18.45% [W/kg] and the power increase is 0.41% [W/kg].

For the ankle joint, Figures 7B,D, the purpose was to assist the plantar-flexion during the push-off phase. The gait cycle instants that report muscular assistance (green areas) are 47% of the whole gait cycle, of which 34% are during the stance and 13% are during the swing. Looking at both the ankle moment and power, it can be seen that assistance occurs in the desired gait cycle phase, during push-off. When the torque and the power report maximum activations, in fact, both the peaks are reduced, but the muscles acting on the joint show noticeable changes in the activation patterns. The corresponding curve is therefore divided into two sub-phases between 42–60% and 60–73% of the gait cycle, Figures 7B,D. In the first sub-phase, the color is green, the moment is reduced by 14.59% [Nm/kg], and the power by 9.4% [W/kg], underlying the effectiveness of the control strategy adopted to assist the plantar-flexor movement. In the second sub-phase, in contrast, the color is red, but the moment reports a reduction by 0.71% [Nm/kg] while the power by 9.81% [W/kg], remarking the efficacy of the control strategy until the last moment of ground contact. In the other phases the signals show some kinetic parameters increase while wearing the exoskeleton, but, considering that our purpose was to assist specifically the push-off phase, as reported in Section 3.2, a general augmentation in the signals at other instants was expected. Calculating the overall moment gain and loss without distinguishing colors, the values show a 15.65% [Nm/kg] reduction, focused mainly on the push-off phase, and a 10.56% [Nm/kg] increase mainly focused on the first part of the gait cycle. The green color is associated with a gain of 10.78%[Nm/kg] for the moment and 4.32% [W/kg] for the power. The red color is associated with a loss of 6.04%[Nm/kg] for the moment, and a gain of 11.23% [W/kg] for the power, mainly due to the contribution during the push-off phase. The yellow color is associated with a gain of 0.35%[Nm/kg] for the moment and 0.9%[W/kg] for the power. Considering the overall gain and loss, the ankle power reduction is 22.27% [W/kg] and the power augmentation is 5.82% [W/kg].

Referring again to Di Natali et al. (2020), the analysis report individual indices (EMG, torque, power, etc.) that do not take into account the multiple interactions that co-occur during walking, and it is based on a gait analysis performed on 10 consecutive steps. On the contrary, the analysis presented in this article takes into account both muscular and kinetic aspects simultaneously and is based on more than 10,000 steps. Moreover, the results obtained through the use of force plates indicate a peak of assistance during the whole releasing phase, but the indices proposed in this work show that the muscle can report the opposite meaning. Kinetic assistance does not always correspond to muscular assistance and in some of the instants during which the joint is receiving the torque from the actuator the muscle is making a greater effort, and, conversely, it benefits from the actuation in other phases of the gait. For these reasons, performing a complex analysis that considers both muscular and kinetic assistance simultaneously provides indices that better attest to the quality of the control strategy.

In conclusion, generating torque trends at the joints associated with force measurements similar to physiological values allows us to highlight how the interaction with the exoskeleton occurs during walking. This allows us to visualize how the muscle responds (whether assisted or resisted) during the motor pattern, the specific joint torque and power trends, and if the user-generated torque while wearing the exoskeleton is below or above the baseline. This information is useful for the developer to make changes to the system.

## 6. Conclusions

In this paper, we have developed a new methodology to assess the effectiveness and impact of an exoskeleton. This approach reduces the number of instruments required for the experimental apparatus and considers a much higher number of cycles compared to traditional kinetic analysis methods. Therefore, the new method is statistically relevant and has been validated and used to evaluate a quasi-passive actuated exosuit for gait assistance. The results underline the effectiveness of the methodology by providing indices that quantify the assistance in terms of torque and power while taking into account the effects of the actuation at a muscular level.

Future studies will be conducted to: (i) apply the methodology to different joints of the body involving the monitoring of a higher number of muscles; (ii) analyze more complex tasks, also considering the kinematic and muscular synergies that occur during their execution. Moreover, this study builds bases to enable the following future works: (iii) implement a real-time analysis to assess the exoskeleton's performances while the user is executing the task; (iv) use real-time data to improve the control strategy optimization; (v) implement the methodology with an adaptive control instead of fixed control rules; (vi) extension for evaluating exoskeletons for rehabilitation after being validated on pathological patients.

## Data availability statement

The raw data supporting the conclusions of this article will be made available by the authors, without undue reservation.

## Ethics statement

The studies involving human participants were reviewed and approved by Ethics Committee of Liguria, Italy (protocol number: 001/2019) and complied with the Helsinki Declaration. The patients/participants provided their written informed consent to participate in this study.

## Author contributions

VF and CD contributed to the conception and design of the study. VF wrote the first draft of the manuscript. VS, DC, JO, and CD contributed to manuscript revision, read, and approved the submitted version. All authors contributed to the article and approved the submitted version.

## Funding

This work has received funding from the European Union's Horizon 2020 framework program for research and innovation under grant agreement no. 688175.

## Conflict of interest

The authors declare that the research was conducted in the absence of any commercial or financial relationships that could be construed as a potential conflict of interest.

## Publisher's note

All claims expressed in this article are solely those of the authors and do not necessarily represent those of their affiliated organizations, or those of the publisher, the editors and the reviewers. Any product that may be evaluated in this article, or claim that may be made by its manufacturer, is not guaranteed or endorsed by the publisher.
